# T1 Mapping–derived Predictors of Cardiac Remodeling and Fibrosis in Athletes using Advanced Machine Learning Techniques

**DOI:** 10.2174/0115734056421491251209121705

**Published:** 2026-01-13

**Authors:** Qian-Feng Luo, Shuang Long, Tao Liu, Jia-Li Li, Dong Chen, Xi-kui Chen, Jing Chen

**Affiliations:** 1 Department of Radiology, The Affiliated Hospital of Southwest Medical University,25 # Tai Ping Street, Luzhou, Sichuan 646000, China; 2 Department of Radiology, The First People’s Hospital of Neijiang, 1866 # Han 'an Avenue West, Neijiang, Sichuan 641000, China

**Keywords:** Athletes, T1 mapping, Machine learning, Cardiac remodeling, Myocardial fibrosis, Extracellular volume

## Abstract

**Introduction:**

This study aimed to predict the occurrence of cardiac remodeling and /or myocardial fibrosis with machine learning based on T1 mapping of cardiovascular magnetic resonance in athletes.

**Methods:**

A total of 104 athletes and 20 healthy sedentary controls underwent a 3.0T cardiovascular magnetic resonance scan. Cardiac function parameters, T1, and extracellular volume values of 16 segments for the left ventricle were measured, respectively. These parameters were separately compared between athletes and controls, as well as between the positive and negative athlete groups. Four machine learning models were constructed for the prediction of cardiac remodeling and /or myocardial fibrosis.

**Result:**

The most effective model was Gradient Boosting Machine, with an AUC value of 0.899, accuracy of 82.7%, sensitivity of 90.0%, and specificity of 81.0%. The top three important factors were the native T1 value of segment 10, the extracellular volume value of segment 3, and body surface area.

**Discussion:**

The direct reason for the increased native T1 value and ECV observed in our study was CR for athletes, which may reveal the relationship between CR and MF. The so-called physiological CR of athletes has certain potential risks. Furthermore, the limitations of this study are that only male athletes were included, and the sample size of the control group was small. This study was a single-center study, and there were selection biases.

**Conclusion:**

Native T1 and extracellular volume values increased in athletes with cardiac remodeling, which may reveal the relationship between cardiac remodeling and myocardial fibrosis. Early cardiac magnetic resonance imaging is conducted to monitor the myocardial native T1 and ECV values of athletes, assess their risk levels, and guide their subsequent surge planning to reduce the occurrence of adverse cardiovascular events.

## 1 INTRODUCTION

### 1.1 Background

Sudden death of athletes in sports occurs occasionally [1-3]. Although the incidence rate is relatively low, it has become a significant problem, attracting considerable attention in cardiovascular research. These events may result from exercise-induced heart disease that has not been detected, such as myocardial fibrosis (MF), arrhythmogenic cardiomyopathy, and hypertrophic cardiomyopathy [4]. Late gadolinium enhancement (LGE) in athletes indicates myocardial fibrosis, which may lead to a series of adverse cardiac events, including a higher incidence of ventricular arrhythmias, reduced ventricular systolic function, and sudden cardiac death [5].

MF is a complex process involving all components of myocardial tissue. Myocardial tissue damage caused by ischemia (hypoxia), hypertensive overload, or inflammation can trigger MF. MF is usually secondary to cardiac remodeling (CR) in diseases such as valve dysfunction, heart failure, and hypertension. Increasing evidence shows that exercise can also lead to CR and MF [4, 6-8]. Studies indicate that intense endurance exercise causes acute dysfunction of the right ventricle, while left ventricular function may remain largely unaffected. Although short-term recovery seems complete, chronic structural changes and reduced right ventricular function are evident in some highly trained athletes.

CR in athletes is generally regarded as a physiological adaptation to exercise. However, some findings of CR overlap with pathological remodeling, making them difficult to distinguish. Furthermore, enlargement of the heart chambers and thickening of the ventricular walls have been associated with adverse cardiovascular events, such as left atrium (LA) and right ventricle (RV) dilatation and left ventricle (LV) hypertrophy [9]. Therefore, predicting the occurrence of myocardial fibrosis and CR in advance to prevent adverse events will be crucial.

### 1.2 Objectives

Without the need for contrast agents, the T1 mapping sequence of cardiovascular magnetic resonance (CMR) can detect diffuse fibrosis using its native T1 mapping sequence. Its extracellular volume (ECV) values can effectively evaluate the degree and extent of myocardial fibrosis, but T1 mapping can only detect the presence of myocardial fibrosis (MF) and cannot predict its occurrence.

Machine learning is an advanced computer science technique. Computer algorithms are applied to large datasets with numerous variables to identify patterns and make predictions based on these data. Machine learning algorithms are typically used to test inputs and build models in order to make data-driven decisions and predictions [10-12]. Compared with traditional prediction models, machine learning often demonstrates better model generalization and prediction accuracy, especially when handling large amounts of data.

In this paper, four machine learning algorithms-gradient boosting machine (GBM), logistic regression (LR), support vector machine (SVM), and classification and regression tree (CART)-were used to differentiate between athletes with and without cardiac remodeling (CR) and/or MF. Currently, few studies have applied machine learning to T1 mapping parameters for evaluating athletes’ myocardium. Some studies have reported the use of T1 mapping in athletes, such as comparing changes in native T1 and ECV values and evaluating the effects of diffuse or focal myocardial fibrosis [13-16], primarily focusing on pre-existing pathological conditions.

Therefore, the aim of this study was to establish machine learning models to predict the occurrence of CR and/or MF in athletes using CMR, providing a noninvasive method for early screening of MF and CR, which may be related to adverse cardiac events. Additionally, the clinical utility of the models was quantified using decision curve analysis (DCA) to calculate the net benefit for patients at different threshold probabilities. This approach may help athletes adjust the frequency of follow-up examinations.

## 2 METHODS

### 2.1 Participants

This paper is based on a machine learning model using T1 mapping to predict adverse cardiovascular events in athletes. This approach is helpful for the early detection and prevention of such events. The study received approval from the Institutional Review Committee of our hospital (Ref: KY2020123), following the ethical guidelines outlined in the Declaration of Helsinki (2013).

According to AHA/ACC (American Heart Association/Arrhythmias Committee of Council) scientific statement standards [17], we prospectively recruited 104 male athletes [average age 24.0 (22.0, 26.0)] from March 2021 to March 2022 in Luzhou, Sichuan Province, considering the influence of gender on native T1 values [18]. All participants were recruited through advertisements, informed about the study, and provided signed informed consent. These athletes were primarily engaged in weight-bearing exercises, running, swimming, and basketball.

#### 2.1.1 Inclusion Criteria

Aged 18–60, exercising ≥6 hours per week, and having ≥3 years of continuous exercise with a complete exercise record.

#### 2.1.2 Exclusion Criteria

Cessation of exercise for more than 6 months, cardiovascular diseases-especially hypertrophic cardiomyopathy (defined as interventricular septum thickness >1.3 cm with family history or >1.5 cm without family history), presence of any cardiovascular risk factors, or any other contraindications for CMR.

In addition, we recruited 20 healthy male controls [average age 24 (23, 25)] who had no clinical symptoms, no abnormal results on relevant physical examinations, exercised less than 3 hours per week, and did not engage in continuous exercise for more than six months.

Exercise time, cardiovascular risk factors, family history of cardiovascular diseases, height, weight, blood pressure, heart rate (HR), neck circumference, waistline, and electrocardiogram results were recorded and measured for all subjects before the examination. All participants also agreed to blood collection for hematocrit (HCT) measurement before the examination.

### 2.2 MRI Protocol

CMR imaging was performed using a 3.0T Siemens Magnetom Scanner (Siemens Healthineers, Prisma, Erlangen, Germany). All subjects were instructed not to exercise vigorously or consume coffee or alcohol for 24 hours before the examination. The protocol included the initial scout images, followed by cine steady-state free precession (SSFP) breath-hold sequences in 2-, 3-, and 4-chamber views. Eight to twelve continuous short-axis images were acquired, covering the ventricles from the plane of the mitral and tricuspid valves to the apex of the heart. The imaging parameters were as follows: repetition time (TR) = 66.2 ms; echo time (TE) = 1.46 ms; slice thickness = 6 mm; phases = 25; flip angle (FA) = 55°; echo spacing = 2.7 ms; field of view (FOV) = 340 mm; voxel size = 1.5 mm × 1.5 mm × 6.0 mm.

Ten minutes after injection of the contrast agent (Gd-DTPA, Guangzhou Kangchen Pharmaceutical Co., LTD), consecutive short-axis LGE images (phase-sensitive inversion recovery, PSIR) were obtained by scanning from the base to the apex, slice by slice. No adverse reactions occurred in the subjects after administration. The imaging parameters were as follows: TE/TR/FA = 1.24 ms / 929.60 ms / 55°, slice thickness = 6.0 mm; voxel size = 1.3 mm × 1.3 mm × 6.0 mm; FOV = 340 mm.

Native T1 mapping images of the left ventricular short-axis were obtained at three slices (basal, mid-cavity, apical) using the modified Look-Locker inversion recovery (MOLLI) sequence. The specific parameter values were: TE/TR/FA = 1.12 ms / 314.16 ms / 35°, slice thickness = 8.0 mm; FOV = 360 mm; voxel size = 1.4 mm × 1.4 mm × 8.0 mm. Fifteen minutes after injection of contrast agent (Gd-DTPA, 0.1 mmol/kg, 3 ml/s), the scan was repeated to obtain the enhanced T1 map in the same three slices [16].

### 2.3 Image Analysis

All images were imported into CVI 42 software (Release 5.12.4, Circle Vascular Imaging, Calgary, Canada) for post-processing. Processing was performed separately by two radiologists (Qian-Feng Luo and Tao Liu), each with more than 3 years of experience in CMR diagnosis and analysis and 4 years of cardiac MRI experience.

#### 2.3.1 Cardiac Function and Related Data

The cine sequence images were loaded into the corresponding processing module, and the endocardium and epicardium of the left and right ventricles were semi-automatically delineated by the software in the short-axis view. The radiologists verified the accuracy of the delineation, and any unreasonable segmentations were corrected. This process yielded a series of cardiac function parameters for the LV and RV: end-systolic volume (ESV), end-diastolic volume (EDV), ejection fraction (EF), stroke volume (SV), heart rate (HR), cardiac output (CO), cardiac index (CI), ventricular mass, and the ratio of these parameters to body surface area (BSA). BSA was used to calculate indexed LV/RV parameters (*e.g*., LVESVi, RVESVi, LVEDVi, RVEDVi). BSA was calculated using the Mosteller formula: BSA (m^2^) = [height (cm) × weight (kg) / 3600]^1/2.

#### 2.3.2 T1 Values

Sixteen segments were measured according to AHA guidelines, excluding the apex (segment 17) of the heart [19]. Native T1 images were loaded into the corresponding processing module. Two radiologists manually delineated the endocardium and epicardium of the left ventricle and marked the blood pool and ventricular septal insertion in these three slices. When delineating the left ventricular endocardium and epicardium, care was taken to avoid including papillary muscles and the blood pool to prevent errors, as shown in Fig. (**1a**). Native T1, enhanced T1, and ECV values of the 16 segments were calculated automatically by the software after inputting the participants’ HCT values. Pseudo-color maps of native T1 and ECV are shown in Fig. (**1b** and **c**).

#### 2.3.3 Criteria for Recognition of CR and MF

The long and short diameters of the ventricles were measured at the four-chamber cardiac level at the end of the diastolic period, and the thickness of the ventricular septum and the left ventricular free wall were also measured. These measurements, combined with the ejection fraction of the ventricles, were used as the criteria for CR (defined as three or more of these parameters exceeding the upper limit of the highest reference value) [20]. Two radiologists identified increased myocardial signal as MF on the LGE sequence and classified the types of delayed enhancement as linear, patchy, subendocardial, or transmural, depending on the site and shape of the enhancement. Both radiologists agreed that LGE was present. In cases of disagreement, consensus was reached after discussion. Fig. (**1d**) shows linear LGE in the ventricular septum (blue arrow). Athletes with CR and/or MF were defined as the positive athlete group, while the others were defined as the negative athlete group.

Intraobserver variability was assessed by comparing measurements of the same observer in 20 randomly selected cases with a 4-week interval. Interobserver variability was assessed by two independently experienced, double-blind observers.

### 2.4 Statistical Analysis

Analysis was performed using the statistical software R (version 4.0.2; R Core Team 2020). The Shapiro-Wilk test was used to assess the normality of the data. All data were tested for normality and homogeneity of variance. When the data were normally distributed, they were expressed as “mean ± standard deviation”; when the data were skewed, they were expressed as “median.” The independent samples t-test and Mann-Whitney U-test were used to compare male athletes and male controls, the positive athletes group and negative athletes group, and positive athletes with only CR athletes and the negative athletes group. A *p*-value <0.05 was considered statistically significant. The inter-class correlation coefficient (ICC) was used to test interobserver and intraobserver consistency. Variables with rare missing data (<5%) were imputed using Multivariate Imputation *via* Chained Equations (“mice” package).

We explored feature importance using machine learning models, including GBM, LR, SVM, and CART. Subsequently, the R package “DALEX” was used to explain these four machine learning approaches, and residual distributions were plotted to identify the best model. In addition, the relative importance of explanatory features was examined for further study. Receiver operating characteristic (ROC) curves, along with the corresponding area under the curve (AUC), were used to evaluate performance. Decision curve analysis (DCA) was applied to quantify the clinical utility of the prediction model, calculating net benefit for patients at various threshold probabilities.

## 3 RESULTS

### 3.1 Comparison of Clinical Baselines and Cardiac Function Parameters between Different Groups

The basic characteristics and cardiac function parameters of athletes and healthy sedentary controls, as well as negative and positive athletes, are shown in Table **1**. The controls had lower weight [60.0 (58.0, 63.5)] and body mass index (BMI) [21.2 (20.4, 21.8)] than athletes (p<0.05). However, their heart rate (HR) [79.0 (75.5, 81.3)] was higher than that of the athletes (p<0.05). Athletes had higher LVEDV (169.4 ± 21.9), RVSV (89.4 ± 20.8), and RVEF (0.50 ± 0.05) than controls (p<0.05).

There were 20 (19.2%) athletes in the positive group, including athletes with CR (n=17, 85%) and athletes with MF (n=3, 15%). In these three athletes, MF occurred mainly in the anteroseptal region at the base and mid-cavity (segments 2, 3, 8, and 9), with a line-shaped distribution involving less than 1–2% of the myocardium. There were statistically significant differences in many variables between the positive and negative groups, including weight, BSA, waistline, LVEDV, LVEF, and LVCI. All of these variables were higher in the positive group (p<0.05), as shown in Table **1**.

### 3.2 Comparison of Native T1 and ECV Parameters between Different Groups

The results of the comparison of native T1 and ECV parameters between different groups are shown in Table **2**. Athletes had lower native T1 values in segments 8 and 14 but higher ECV values in segments 1, 4, 6, 8, and 9 compared to controls (p<0.05). Positive athletes had higher native T1 values in segments 3, 6, 8, 9, 10, 14, and 15 and higher ECV values in segments 3, 6, and 8 than negative athletes (p<0.05). The specific values are detailed in Table **2**.

To exclude the effect of MF in the positive group, we analyzed the differences in native T1 and ECV values between the remaining positive athletes and the negative athletes group. The results are shown in Table **3**. Positive athletes with only CR had higher native T1 values in segments 3, 9, 10, 14, and 15 and higher ECV values in segments 3, 6, 8, and 9 (p<0.05).

### 3.3 Prediction Models for Cardiac Remodeling and/or MF with Machine Learning

As shown in Fig. (**2**), the ranking of AUC values for the four models from high to low was GBM (0.899), LR (0.842), SVM (0.812), and CART (0.716). The performance of the four models is summarized in Table **4**. DCA is shown in Fig. (**3**); when the threshold probability was between 0 and approximately 0.24, the GBM model had the highest net benefit and best clinical efficacy (AUC 0.899, accuracy 82.7%, sensitivity 90.0%, and specificity 81.0%). Therefore, GBM was chosen as the best-performing model.

The GBM model included many features, such as baseline variables (BSA, weight, and waistline), native T1 values of segments 3, 6, 8, 9, 10, 14, and 15, and ECV values of segments 3, 6, and 8. The relative importance of these features is shown in Fig. (**4**). Among them, the top six features, ranked from highest to lowest, were: native T1 value of segment 10, ECV value of segment 3, BSA, weight, native T1 value of segment 3, and native T1 value of segment 6.

### 3.4 Reproducibility of Native T1 and ECV Values

The ICC values of native T1 and ECV for intraobserver and interobserver measurements were calculated, and all segments were greater than 0.75, indicating excellent consistency. The range of ICCs for intraobserver and interobserver native T1 values was 0.757–0.971 and 0.760–0.984, respectively. The range for intraobserver and interobserver ECV values was 0.882–0.984 and 0.854–0.978, respectively.

## 4 DISCUSSION

### 4.1 Main Finding

In this study, exercise-induced CR and MF were found to alter myocardial native T1 and ECV values, which are associated with myocardial fibrosis according to previous studies [21]. However, we found that the direct cause of the increased native T1 and ECV values in athletes was CR, which has not been mentioned in previous literature and may reveal the relationship between CR and MF. The so-called physiological CR in athletes may have certain hidden risks. In addition, we used the GBM model, which demonstrated high efficiency, to predict CR and/or MF, potentially associated with adverse outcomes. Early detection may help draw attention to exercise practices, increase the frequency of CMR follow-up, and help prevent adverse outcomes. During follow-up, specific regions of the left ventricular wall should be focused on, including the basal inferoseptal wall (segment 3), mid-cavity inferior wall (segment 10), and basal anterolateral wall (segment 6).

### 4.2 The Changes of Native T1 and ECV Parameters by Exercise

Athletes with MF were reported to have increased native T1 and ECV values of the LV and a higher incidence of ventricular arrhythmias and adverse cardiac outcomes [22-24]. Excluding the influence of MF in this study, athletes with only CR also showed increased native T1 and ECV values, indicating that CR may be the cause of this increase and suggesting a possible relationship between CR and MF. Myocardial fibrosis is defined as a significant increase in the volume of collagen in cardiac tissue [25]. It is a complex and long-term process. When CR occurs, myocardial cells may be damaged, but the volume of collagen in cardiac tissue may not yet reach the level of myocardial fibrosis. In this context, the T1 mapping sequence can detect changes in native T1 and ECV values, whereas the conventional LGE sequence cannot. Another possibility is that cardiac remodeling leads to increased myocardial space and alterations in certain substances within the interstitium, which should be further investigated through pathological biopsies in the future. Native T1 and ECV values are therefore expected to serve as indicators for evaluating the relationship between CR and myocardial fibrosis and may be used to quantify critical values or ranges of cardiac remodeling and myocardial fibrosis for risk stratification in the future.

### 4.3 Clinical Application of the GBM Model

In this study, we ultimately chose GBM as the best model because it provided the highest net benefit and the best clinical efficacy. Among the variables ranked by relative importance in this model, the native T1 or ECV values of segments 3, 6, and 10 were the most important. We will discuss why these variables were selected. Although the coronary blood supply to different myocardial segments is variable, it is reasonable to assign individual segments to specific coronary areas [19]. Segments 3 and 10 play an important role in this model, as they are mainly supplied by the RCA. Some studies have shown that endurance exercise causes RV remodeling [7, 9, 26, 27].

Increased oxygen consumption and biventricular cardiac output during exercise are associated with reduced resistance in the pulmonary circulation. This mismatch between increased flow and vasodilation leads to inappropriate rises in pulmonary artery pressure and RV afterload. At rest, the RV maintains an appropriate atrioventricular pressure gradient. However, during exercise, the atrioventricular valve is closed, and the high flow state results in massive atrial filling and elevated systolic pressure. This higher pressure in the LA supports pulmonary circulation but increases RV afterload, leading to RV dilation.

We hypothesize that, with RV dilation and an unchanged number of cardiomyocytes, the extracellular space, composed of plasma and extravascular interstitial space [28], expands, thereby increasing the blood supply required by the RCA. This expansion affects the small branches of the RCA that supply segments 3 and 10. At rest, a slight decrease in LVEF and reduced coronary blood flow further compromise RCA perfusion to segments 3 and 10. In T1 mapping sequences, ECV values increase with interstitial space expansion. Due to the dilated ventricles having increased volume and contractile reserve [27], these changes often produce no obvious clinical symptoms and cannot be detected by conventional CMR. However, long-term reduced blood supply leads to myocardial cell damage (myocardial fibrosis), which can be detected as abnormalities on T1 mapping sequences. This is likely a slow process influenced by factors such as exercise intensity, frequency, and individual differences.

Segment 6, defined as the remote myocardium (180° from segment 3) [16], rarely develops myocardial fibrosis. In contrast, segment 3 (the inferoseptal segment) appears more prone to fibrosis in endurance athletes [27]. Consequently, the native T1 and ECV values of segment 3 significantly increase, showing strong comparability and likely explaining why it stands out among the variables.

BSA and weight were also among the variables with high relative importance identified by the GBM model, with BSA being calculated from height and weight.

Due to individual differences, the weight and BSA of subjects are not the same, which can better reflect the real situation of individuals. Being overweight is also a risk factor for cardiometabolic diseases, such as type 2 diabetes and cardiovascular disease. These pathological conditions lead to a deterioration in quality of life and an increased risk of death [29-31]. Left ventricular hypertrophy and left ventricular dilatation are well-established risk factors for cardiovascular disease in adults and have been shown in one study to be reversible after weight loss surgery in a small number of subjects [32]. The high importance of these two variables indicates the rationality of the GBM model.

At present, few studies have applied machine learning models to predict CR and MF in athletes, especially based on T1 mapping parameters of all 16 segments of the left ventricle, which could reflect focal sensitive segments better than global left ventricular wall parameters. In addition, the GBM model has the best comprehensive efficiency (AUC, accuracy, sensitivity, specificity). The model synthetically considers myocardial native T1 values and ECV values from multiple perspectives, along with some baseline data, to make predictions more individualized for subjects. For athletes with high physical activity, predicting the occurrence of CR and MF facilitates early follow-up and monitoring. Moreover, the T1 mapping sequence has a very short scan time, which is conducive to myocardial screening and follow-up.

## 5 LIMITATIONS

Firstly, this study only included male athletes because most sudden cardiac deaths occur in male athletes, and there are gender differences in native T1 values between males and females [18]. In addition, male athletes are more common than female athletes. We plan to include female athletes for comparison in a future study. Secondly, this study is a single-center study, which may introduce selection bias. A multi-center study is planned. Thirdly, the sample size of this study is small, especially in the control group, because few healthy individuals are willing to undergo enhancement scanning. Therefore, the generalizability of the model needs to be further verified. The GBM model was developed and tested on a single dataset without external validation, which may limit its applicability to other populations or imaging protocols. More subjects should be included in future studies to validate this model. Fourth, this study is cross-sectional and cannot establish a causal relationship between cardiac remodeling and myocardial fibrosis. Finally, the MF observed in positive athletes may be reversible, and follow-up examinations over time are needed to determine whether it decreases or progresses.

## CONCLUSION

Not only athletes with MF, but also those with CR, can exhibit increased native T1 and ECV values, suggesting a relationship between CR and myocardial fibrosis, and indicating that CR may be a prelude to MF. In this study, a GBM model with higher diagnostic efficiency was successfully constructed based on T1 mapping sequence parameters to predict the occurrence of CR and/or MF, which may be associated with adverse cardiac events, and is helpful for adjusting the frequency of follow-up and monitoring of athletes. The native T1 values and ECV values of the basal inferoseptal wall (segment 3), mid-cavity inferior wall (segment 10), and basal anterolateral wall (segment 6) were emphasized during the follow-up process.

## Figures and Tables

**Fig. (1):**
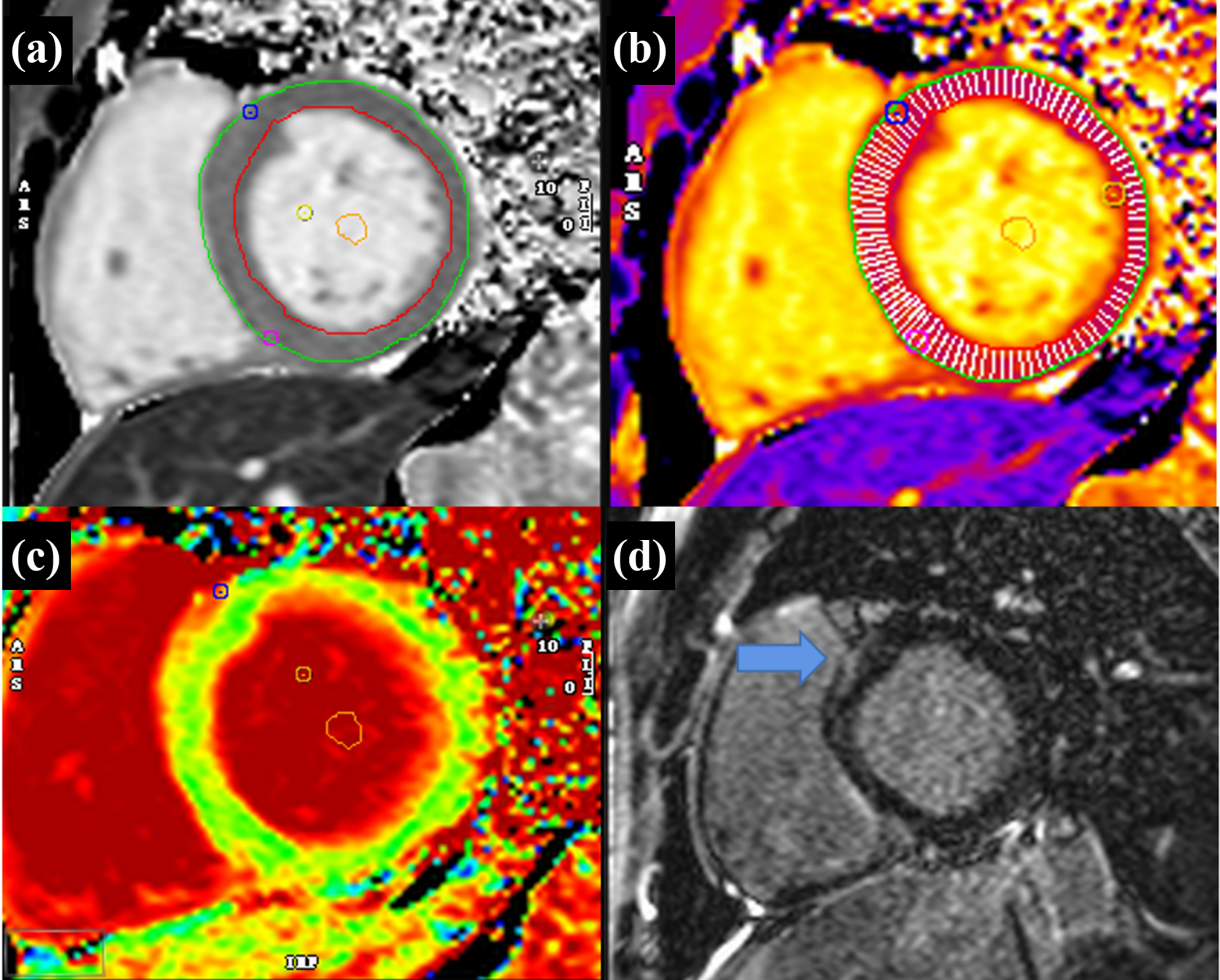
Four images related to T1 mapping of a male athlete, 24 years old with LGE. (**a**): The endocardium and epicardium are manually delineated and the blood pool and ventricular septum are marked. (**b**): Native T1 pseudo-color map. (**c**): ECV pseudo-color map. (**d**): Late gadolinium enhancement imaging, showing linear high signal in basal anteroseptal segment (blue arrow). All images come from the basal. ECV, extracellular volume.

**Fig. (2):**
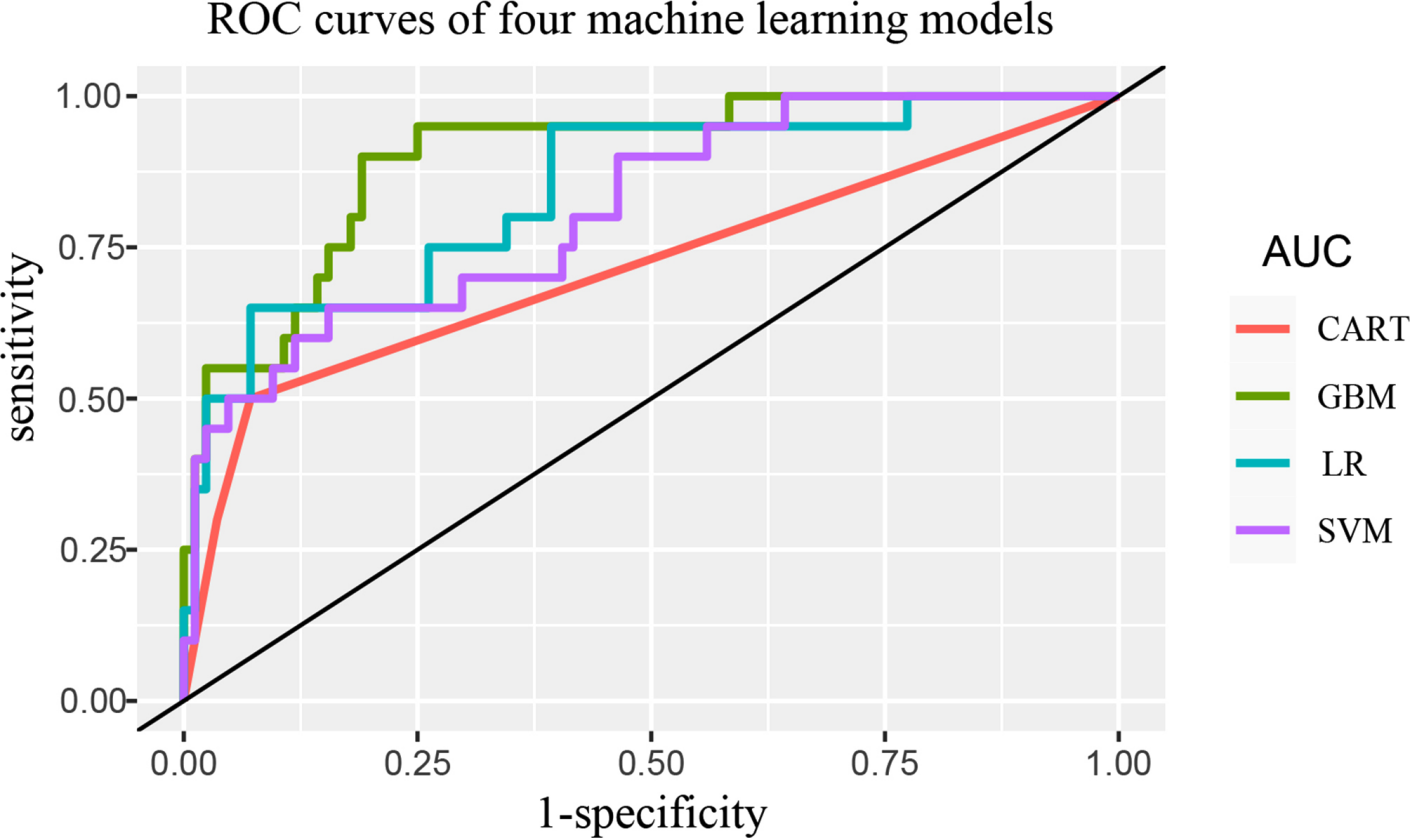
ROC curves of four machine learning models. X-axis is the false positive rate of models, and Y-axis is the true positive rate of models. It can be seen that GBM has the highest AUC. ROC, Receiver operating characteristic; AUC, area under the curve; GBM, Gradient boosting machines; LR, logistic regression; SVM, supported vector machine; CART, classification and regression tree.

**Fig. (3):**
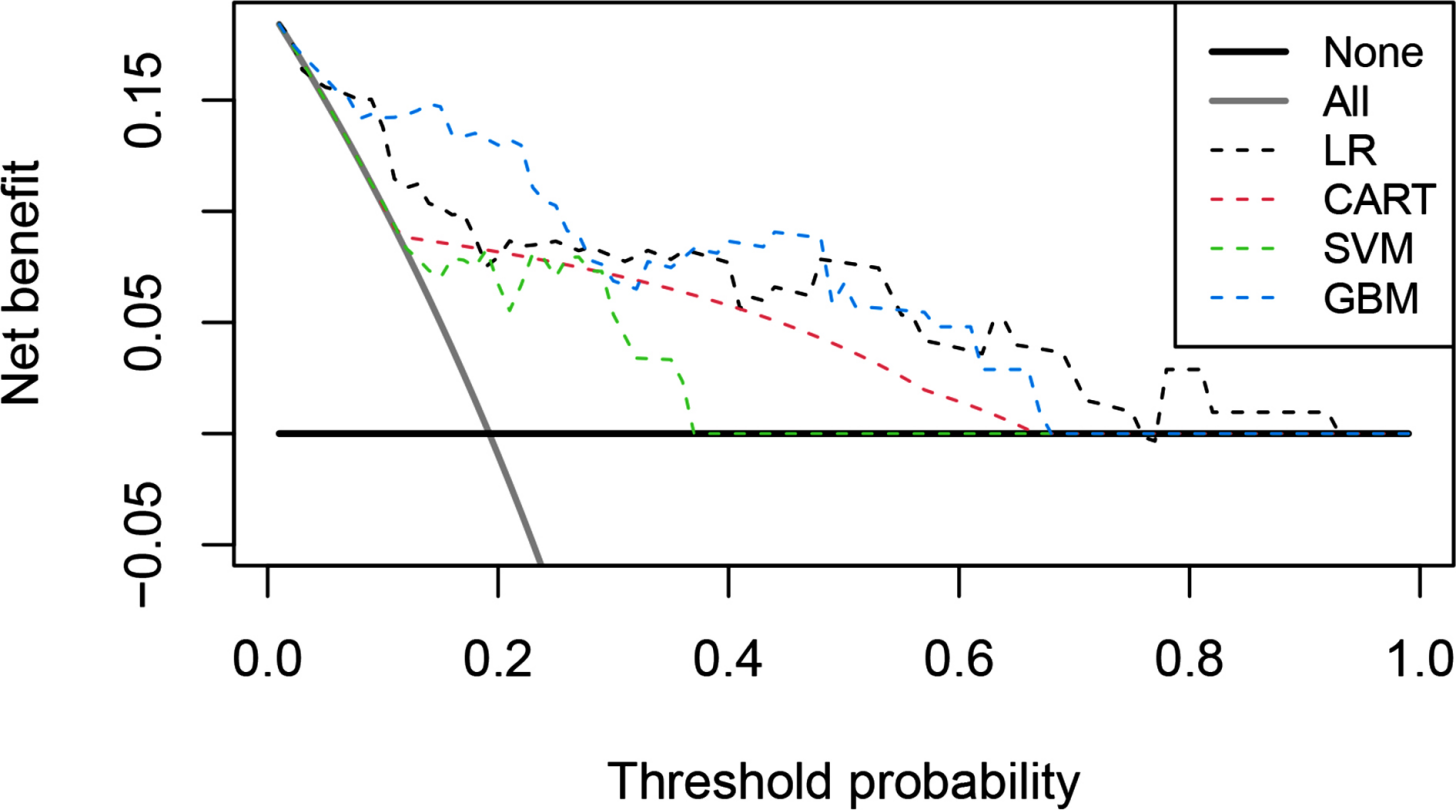
Decision curve analysis of four machine learning models. X-axis is the threshold probability of models, and Y-axis is the net benefit of models. When the threshold probability was between 0 and about 0.24, the GBM model had the best net benefit and the best clinical efficacy. GBM, Gradient boosting machines.

**Fig. (4):**
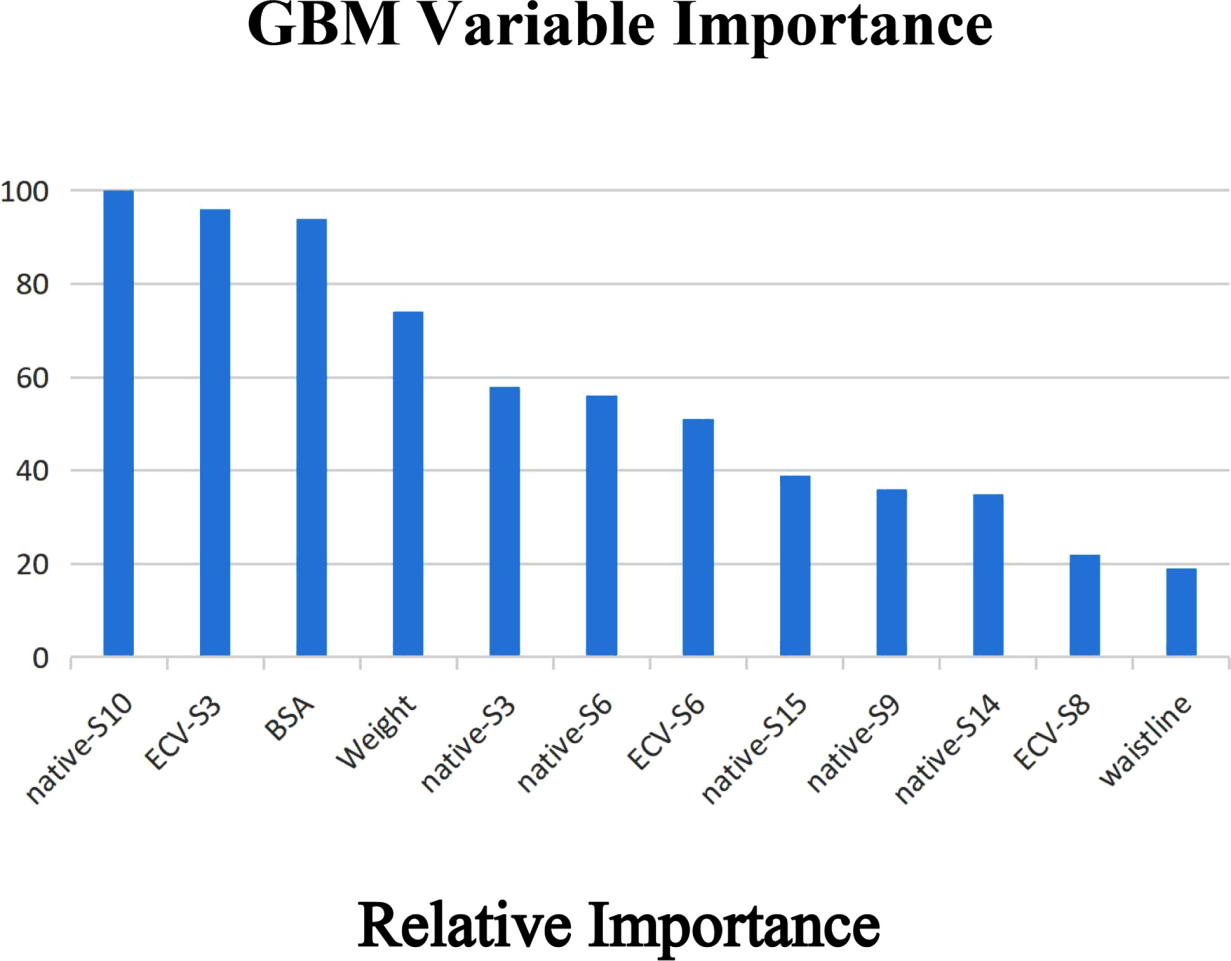
Variable Importance of GBM.X-axis is the different variables of models, and Y-axis is the relative importance of models. A series of variables were filtered out, the top five predictors from high to low was as follows: native T1 value of 10^th^ segment, ECV value of 3rd segment, BSA, weight and native T1 value of 3^rd^ segment. GBM, Gradient boosting machines; ECV, extracellular volume; BSA, body surface area.

**Table 1:** Baselines and cardiac function parameters of athletes and controls, negative athletes and positive athletes.

**Variables**	**Controls (n = 20)**	**athletes (n = 104)**	***p*-value**	**negative athletes (n = 84)**	**positive athletes (n = 20)**	***p*-value**
Age	24 (23, 25)	24 (22, 26)	0.822	24 (22, 25.2)	25 (22, 27)	0.344
Height	170.0 (168.8, 175.0)	174.0 (170.0, 177.0)	0.071	173.0 (170.0, 176.2)	175.0 (173.0, 178.5)	0.093
Weight	60.0 (58.0, 63.5)	68.5 (64.0, 74.0)	**< 0.001**	67.0 (62.9, 73.0)	72.0 (68.8, 81.5)	**0.004**
BMI	21.2 (20.4, 21.8)	22.7 (21.4, 24.5)	**< 0.001**	22.6 (21.2, 24.2)	23.8 (22.0, 25.6)	0.108
BSA	1.8 ± 0.1	1.9 ± 0.1	**< 0.001**	1.9 (1.8, 2.0)	2 (1.9, 2.0)	**0.004**
Neck circumference	35.0 (33.8, 37.3)	36.0 (35.0, 38.0)	0.055	36.0 (35.0, 38.0)	37.0 (36.0, 38.8)	0.245
Waistline	79.0 (75.8, 82.1)	81.8 (77.0, 86.0)	0.073	81.0 (76.0, 85.0)	83.5 (80.8, 88.0)	**0.047**
Years of exercise		5 (4, 8)		5 (4, 8)	4 (4, 8.5)	0.646
SBP	123.8 ± 8.8	119.9 ± 11.9	0.102	119.3 ± 11.8	122.2 ± 12.1	0.332
DBP	79.4 ± 9.7	76.0 ± 9.4	0.162	75.6 ± 9.6	76.7 ± 10.0	0.647
HR	79.0 (75.5, 81.3)	67 .0(61.0, 75.5)	**< 0.001**	67 (61.0, 75.0)	65.5 (61.8, 78.5)	0.931
LVEDV	147.4 ± 20.4	169.4 ± 21.9	**< 0.001**	165.0 ± 19.6	189.0 ± 20.9	**<0.001**
LVESV	63.6 ± 12.8	66.8 ± 15.9	0.334	67.4 ± 16.7	64.2 ± 12.3	0.335
LVSV	88.2 ± 15.5	92.5 ± 19.9	0.283	92.1 ± 20.8	94.2 ± 15.5	0.617
LVEF	0.58 ± 0.06	0.58 ± 0.05	0.979	0.58 ± 0.05	0.60 ± 0.03	**0.03**
LVCO	5.95 ± 0.92	5.83 ± 1.12	0.622	5.76 ± 1.17	6.12 ± 0.83	0.127
LVCI	3.45 ± 0.52	3.26 ± 0.51	0.155	3.22 ± 0.54	3.43 ± 0.32	**0.033**
LVM	96.7 ± 20.6	100.7 ± 21.7	0.435	99.8 ± 22.1	104.0 ± 19.9	0.4
LVEDVi	87.8 ± 10.7	88.8 ± 14.0	0.738	88.8 ± 14.6	88.6 ± 11.5	0.935
LVESVi	36.84 ± 7.11	37.18 ± 7.26	0.844	37.5 ± 7.65	35.8 ± 5.23	0.251
SVi	51.01 ± 8.09	51.59 ± 9.04	0.774	51.3 ± 9.46	52.7 ± 7.09	0.456
RVEDV	174.0 ± 22.7	177.97 ± 38.1	0.536	178 ± 39.5	179 ± 32.5	0.935
RVESV	92.9 ± 16.6	88.6 ± 21.4	0.316	89.0 ± 21.8	86.6 ± 20.1	0.64
RVSV	81.2 ± 12.1	89.4 ± 20.8	**0.019**	88.8 ± 21.6	91.9 ± 17.7	0.505
RVEF	0.47 ± 0.05	0.50 ± 0.05	**0.011**	0.50 ± 0.05	0.52 ± 0.05	0.197
RVCO	5.48 ± 0.78	5.65 ± 1.28	0.432	5.57 ± 1.31	5.99 ± 1.14	0.163
RVCI	3.18 ± 0.42	3.16 ± 0.62	0.88	3.11 ± 0.64	3.35 ± 0.50	0.075
RVEDVi	100.8 ± 11.6	99.1 ± 16.6	0.592	99.0 ± 17.4	99.6 ± 13.0	0.863
RVESVi	53.8 ± 9.4	49.3 ± 9.8	0.058	49.5 ± 10.1	48.1 ± 8.5	0.531
RVSVi	46.9 ± 6.0	49.8 ± 9.8	0.083	49.5 ± 10.1	51.4 ± 8.3	0.362

**Note:**Values are Mean ± SD or Median (Q1,Q3). Values in bold indicate significant differences between groups. BMI, Body mass index; BSA, body surface area; SBP, Systolic blood pressure; DBP, Diastolic blood pressure; HR, Heart rate; LV, left ventricle; EDV, end-diastolic volume; ESV, end-systolic volume; SV, stroke volume; EF, ejection fraction; CO, cardiac output; CI, cardiac index; LVM, left ventricle mass; EDVi, end-diastolic volume index; ESVi, end-systolic volume index; SVi, stroke volume index. RV, right ventricle.

**Table 2:** Native T1 and ECV parameters of athletes and controls, positive and negative athletes groups.

**Variables**	**Controls (n = 20)**	**athletes (n = 104)**	***p*-value**	**negative athletes (n = 84)**	**positive athletes (n = 20)**	***p*-value**
Native-S1	1180 (1157, 1215)	1188 (1156, 1208)	0.686	1187 (1156, 1206)	1190 (1174, 1210)	0.417
Native-S2	1239 ± 38	1223 ± 32	0.09	1222 ± 32	1230 ± 30	0.272
Native-S3	1229 ± 44	1222 ± 30	0.5	1218 ± 30	1236 ± 25	**0.012**
Native-S4	1215 ± 50	1206 ± 35	0.424	1203 ± 36	1217 ± 32	0.1
Native-S5	1185 (1153, 1197)	1173 (1150, 1193)	0.47	1172 (1149, 1192)	1176 (1150, 1204)	0.635
Native-S6	1186 (1163, 1212)	1170 (1144, 1196)	0.114	1168 (1140, 1191)	1186 (1173, 1206)	**0.048**
Native-S7	1200 (1165, 1223)	1183 (1163, 1208)	0.189	1183. (1162, 1208)	1177 (1167, 1204)	0.895
Native-S8	1227 ± 24	1210 ± 36	**0.014**	1206 ± 36	1225 ± 32	**0.033**
Native-S9	1226 ± 23	1216 ± 31	0.097	1213 ± 31	1231 ± 30	**0.021**
Native-S10	1195 (1169, 1227)	1186 (1155, 1211)	0.143	1185 (1147, 1199)	1206 (1187, 1224)	**0.004**
Native-S11	1148 (1138, 1175)	1151 (1120, 1182)	0.611	1143 ± 47	1160 ± 47	0.161
Native-S12	1204 (1157, 1230)	1186 (1167, 1216)	0.397	1186 (1166, 1216)	1188 (1170, 1206)	0.833
Native-S13	1217 (1191, 1235)	1203 (1171, 1235)	0.204	1203 (1172, 1237)	1202 (1165, 1224)	0.745
Native-S14	1231 ± 25	1214 ± 38	**0.016**	1208 ± 37	1235 ± 40	**0.012**
Native-S15	1196 ± 40	1179 ± 37	0.087	1174 ± 37	1198 ± 29	**0.003**
Native-S16	1194 (1176, 1214)	1190 (1164, 1213)	0.539	1194 ± 47	1187 ± 41	0.496
ECV-S1	0.23 (0.21, 0.25)	0.24 (0.23, 0.26)	**0.048**	0.24 (0.22, 0.26)	0.24 (0.24, 0.25)	0.069
ECV-S2	0.24 ± 0.03	0.25 ± 0.02	0.142	0.25 ± 0.02	0.26 ± 0.01	0.059
ECV-S3	0.24 ± 0.03	0.25 ± 0.02	0.061	0.25 ± 0.02	0.26 ± 0.02	**0.024**
ECV-S4	0.24 ± 0.03	0.25 ± 0.02	**0.046**	0.25 ± 0.03	0.26 ± 0.02	0.823
ECV-S6	0.22 (0.2, 0.24)	0.24 (0.22, 0.25)	**0.002**	0.24 (0.22, 0.25)	0.25 (0.23, 0.25)	**0.045**
ECV-S8	0.24 ± 0.02	0.26 ± 0.02	**0.048**	0.25 ± 0.02	0.26 ± 0.02	**0.042**
ECV-S9	0.24 ± 0.03	0.25 ± 0.02	**0.042**	0.25 ± 0.02	0.26 ± 0.02	0.068
ECV-S10	0.24 ± 0.03	0.25 ± 0.02	0.192	0.25 ± 0.02	0.25 ± 0.02	0.178
ECV-S16	0.24 (0.23, 0.29)	0.26 (0.24, 0.28)	0.127	0.26 ± 0.03	0.26 ± 0.03	0.585

**Note:**Values are Mean ± SD or Median (Q1,Q3). Values in bold indicate significant differences between groups. ECV, Extracellular volume; Native-S1, native T1 values of segment 1; ECV-S1, Extracellular volume values of segment 1, by that analogy. The native T1 values of most segments of the athletes were lower than those of the control group, with statistically significant differences in segments 8 and 14. The ECV values of most segments of the athletes were higher than those of the control group, and there were statistically significant differences in segments 1,4,6,8, and 9. Positive athletes had higher native T1 values of segments 3,6,8,9,10,14 and 15 and ECV values of segments 3,6 and 8 than negative athletes.

**Table 3:** Native T1 and ECV values between negative athletes and positive athletes only with CR.

**Variables**	**Negative athletes(n=84)**	**Only CR athletes(n=17)**	***p*-value**
Native-S1	1187 (1156, 1206)	1188 (1170, 1208)	0.592
Native-S2	1222 ± 32	1230 ± 32	0.355
Native-S3	1218 ± 30	1239 ± 25	**0.006**
Native-S4	1203 ± 36	1218 ± 34	0.117
Native-S5	1173 (1150, 1192)	1172 (1150, 1202)	0.897
Native-S6	1168 (1140, 1191)	1184 (1175, 1205)	0.097
Native-S7	1184 (1162, 1208)	1169 (1163, 1201)	0.601
Native-S8	1209 (1187, 1232)	1220 (1200, 1264)	0.059
Native-S9	1213 ± 31	1233 ± 32	**0.028**
Native-S10	1185 (1147, 1199)	1213 (1182, 1226)	**0.009**
Native-S11	1144 ± 47	1163 ± 46	0.136
Native-S12	1186 (1166, 1216)	1186 (1169, 1201)	0.989
Native-S13	1204 (1173, 1237)	1201 (1156, 1229)	0.683
Native-S14	1209 ± 36	1238 ± 43	**0.019**
Native-S15	1174 ± 37	1201 ± 30	**0.003**
Native-S16	1194 ± 47	1189 ± 41	0.659
ECV-S1	0.24 (0.22, 0.26)	0.24 (0.24, 0.25)	0.057
ECV-S2	0.25 ± 0.02	0.26 ± 0.02	0.063
ECV-S3	0.25 ± 0.02	0.26 ± 0.01	**0.006**
ECV-S4	0.25 (0.24, 0.27)	0.26 (0.25, 0.27)	0.141
ECV-S6	0.24 (0.22, 0.25)	0.25 (0.23, 0.26)	**0.029**
ECV-S8	0.25 ± 0.02	0.27 ± 0.02	**0.019**
ECV-S9	0.25 ± 0.02	0.26 ± 0.02	**0.029**
ECV-S10	0.25 ± 0.02	0.26 ± 0.02	0.064
ECV-S16	0.26 ± 0.03	0.26 ± 0.03	0.966

**Note:**Values are Mean ± SD or Median (Q1,Q3). Values in bold indicate significant differences between groups. ECV, Extracellular volume; Native-S1, native T1 values of 1^st^ segment; ECV-S1, Extracellular volume values of 1^st^ segment, by that analogy.

**Table 4:** Efficiency of the models.

**Model**	**AUC**	**Accuracy**	**Sensitivity**	**Specificity**
GBM	0.899	0.827	0.9	0.81
LR	0.842	0.875	0.65	0.929
SVM	0.812	0.808	0.65	0.845
CART	0.716	0.846	0.5	0.929

**Note:**AUC, area under the curve; GBM, Gradient boosting machines; LR, logistic regression; SVM, supported vector machine; CART, classification and regression tree. GBM model has the highest AUC and sensitivity, and its accuracy and specificity also perform well(greater than 0.8).

## Data Availability

The data and supportive information are available within the article.

## LIST OF ABBREVIATIONS

MF: Myocardial Fibrosis
LGE: Late Gadolinium Enhancement
CR: Cardiac Remodeling
CMR: Cardiovascular Magnetic Resonance
ECV: Extracellular Volume
GBM: Gradient Boosting Machine
LR: Logistic Regression
SVM: Support Vector Machine

## STANDARDS OF REPORTING

STROBE guidelines were followed.
